# The complete chloroplast genome sequence of *Celtis tetrandra*

**DOI:** 10.1080/23802359.2019.1674705

**Published:** 2019-10-11

**Authors:** Yi Wang, Xiaolong Yuan, Jinfeng Zhang

**Affiliations:** Laboratory of Forest Plant Cultivation and Utilization, Yunnan Academy of Forestry, Kunming, Yunnan, People's Republic of China

**Keywords:** *Celtis tetrandra*, chloroplast, Illumina sequencing, phylogenetic analysis

## Abstract

The first complete chloroplast genome sequences of *Celtis tetrandra* were reported in this study. The cpDNA of *C. tetrandra* is 159,014 bp in length, contains a large single-copy region (LSC) of 86,097 bp, and a small single-copy region (SSC) of 19,129 bp, which were separated by a pair of inverted repeat (IR) regions of 26,894 bp. The genome contains 131 genes, including 86 protein-coding genes, 8 ribosomal RNA genes, and 37 transfer RNA genes. The overall GC content of the whole genome is 36.3%. Phylogenetic analysis of 10 chloroplast genomes within the family Cannabaceae suggests that *C. tetrandra* clustered together with *Celtis biondii* in a unique clade.

Cannabaceae includes ten genera that are widely distributed in tropical to temperate regions of the world (Yang et al. 2013). *Celtis tetrandra* [synonymous with *Celtis salvatiana* C. K. Schneid. *Celtis kunmingensis* W. C. Cheng et T. Hong] belongs to the genus *Celtis* in Cannabaceae and is a wild deciduous arbour (8–20 m high), is distributed mainly in Yunnan, China and also distributed in Southeast Asia (Xu 1990). *Celtis tetrandra* is an important indigenous and greening tree species in Yunnan (Feng 2018). The extract from the bark of *C. tetrandra* also showed TRAIL resistance-overcoming activity (Seephonkai et al. 2018). However, there have been no genomic studies on *C. tetrandra*.

Herein, we reported and characterized the complete *C. tetrandra* plastid genome (MN017129). One *C. tetrandra* individual (specimen number: 201804019) was collected from Kunming botanical garden, Kunming, Yunnan Province of China (25°14′18″N, 102°75′12″E). The specimen is stored at Yunnan Academy of Forestry Herbarium, Kunming, China, and the accession number is YAFH0012867. DNA was extracted from its fresh leaves using DNA Plantzol Reagent (Invitrogen, Carlsbad, CA, USA).

Paired-end reads were sequenced by using Illumina HiSeq system (Illumina, San Diego, CA). In total, about 29.4 million high-quality clean reads were generated with adaptors trimmed. Aligning, assembly, and annotation were conducted by CLC de novo assembler (CLC Bio, Aarhus, Denmark), BLAST, GeSeq (Tillich et al. 2017), and GENEIOUS v 11.0.5 (Biomatters Ltd, Auckland, New Zealand). To confirm the phylogenetic position of *C. tetrandra*, other 9 species of family Cannabaceae from NCBI were aligned using MAFFT v.7 (Katoh and Standley 2013) and maximum likelihood (ML) bootstrap analysis was conducted using RAxML (Stamatakis 2006); bootstrap probability values were calculated from 1000 replicates. *Debregeasia orientalis* (MH196364) and *Cecropia pachystachya* (MF953831) were served as the out-group.

The complete *C. tetrandra* plastid genome is a circular DNA molecule with the length of 159,014 bp, with a large single copy (LSC: 86,097 bp), small single copy (SSC: 19,129 bp), and two inverted repeats (IRa and IRb: 26,894 bp each). The overall GC content of the whole genome is 36.3%, and the corresponding values of the LSC, SSC, and IR regions are 34.0, 29.9, and 42.3%, respectively. The genome contains 131 genes, including 86 protein-coding genes, 8 ribosomal RNA genes, and 37 transfer RNA genes. Phylogenetic analysis showed that *C. tetrandra* clustered together with *Celtis biondii* in a unique clade, which indicated the phylogenesis classification of *C. tetrandra* in family Cannabaceae (Figure 1). The determination of the complete plastid genome sequences provided new molecular data to illuminate the Cannabaceae evolution.

**Figure 1.**
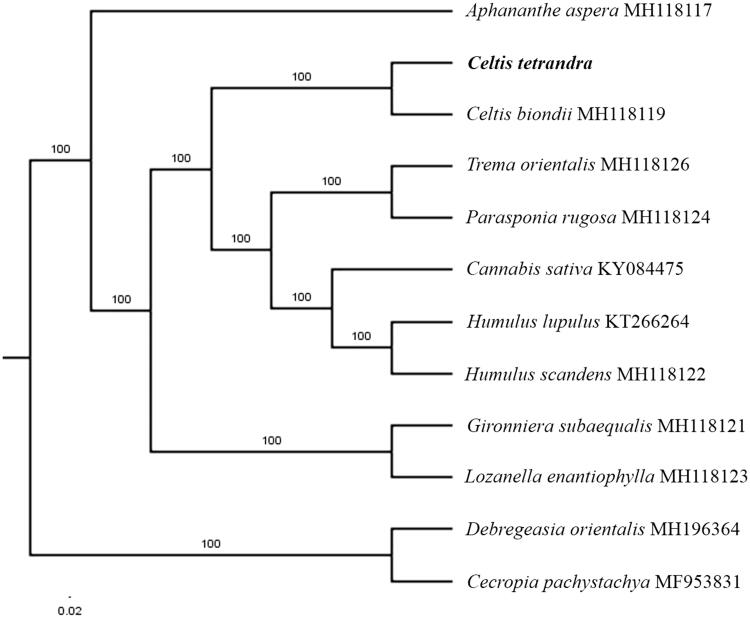
The maximum-likelihood tree based on the 10 chloroplast genomes of family Cannabaceae. The bootstrap value based on 1000 replicates is shown on each node.
